# TELEWORKING TO SUPPORT ACCOMMODATION, INCLUSION, AND HEALTH OF OLDER WORKERS: ISSUES FACED BY MANAGERS

**DOI:** 10.1093/geroni/igad104.3567

**Published:** 2023-12-21

**Authors:** Alexandra Lecours

**Affiliations:** Université du Québec à Trois-Rivières, Drummondville, Quebec, Canada

## Abstract

Telework is increasingly present and has the potential to be used as an accommodation modality to facilitate inclusion and healthy participation in the workplace for older workers (i.e. aged 55 and over). However, there is a need for a practical tool to guide the application of telework with this population. This poster presents the first stage of a study aiming to develop a reflective guide for applying telework to support the accommodation, inclusion, and health of older workers. Following a three-stage developmental research design, this first stage consists in conducting individual interviews with older teleworkers and managers to gather qualitative data on their experience. During the second stage, the issues, considerations, and good practices that emerged from the first stage are compiled into a guide. In the third stage, this guide will be validated by workers and managers to ensure its acceptability and applicability. Preliminary results from the first stage allowed to identify issues faced by managers concerning accommodation (n=8, e.g. ensuring that accommodating older workers does not add extra pressure on colleagues), inclusion (n=5, e.g. difficulty to mobilize older teleworkers for face-to-face social gatherings), and health (n=2, e.g. managers feel powerless to manage older teleworkers’ emotions). Managers play a key role in implementing practices that promote the accommodation and inclusion of older workers through teleworking, but they have few tools to support them. The reflective application guide that will be created during this study represents an innovative tool likely to have positive impacts at individual, organizational and societal levels.

